# Glycopyrrolate for drooling in children with medical complexity under three years of age

**DOI:** 10.1186/s13052-021-01195-1

**Published:** 2022-01-08

**Authors:** Eleonora Lovardi, Maria Antonietta De Ioris, Donatella Lettori, Caterina Geremia, Susanna Staccioli, Gessica Della Bella, Raffaella Scrocca, Alessia Scarselli, Marcella Aversa, Francesco De Peppo, Andrea Campana, Enrico Castelli

**Affiliations:** 1 Neurorehabilitation Unit, Department of Neurosciences, Bambino Gesù Children Hospital, IRCCS, Rome, Italy; 2 Child Neuropsychiatric Unit, Department of Systems Medicine, Tor Vergata University of Rome, Rome, Italy; 3 Pediatrics, University Department of Pediatrics, Rome, Italy; 4 Intensive Care Unit, Department of Anesthesia and Intensive Care, Rome, Italy; 5 Department of Pediatric Surgery and Transplantation Center, Bambino Gesù Children Hospital, IRCCS, Rome, Italy

**Keywords:** Drooling, Swallowing, Children with medical complexity, Neurodevelopmental disabilities, Case report

## Abstract

**Background:**

The aim of the study is to determine that Glycopirrolate is safe and effective in decreasing drooling in children with medical complexity under 3 years of age. Medical treatment is based on anticholinergic drugs as transdermal scopolamine, benzotropine and GLY. GLY (Glycopyrronium bromide) is a synthetic quaternary ammonium anticholinergic agent with poor blood–brain barrier penetration and consequently has limited central effects. Actually, the oral GLY formulation was approved by the United States Food and Drug Administration (FDA) to treat drooling in children aged 3–16 years. Five studies reported on GLY use for the treatment of drooling in children with cerebral palsy and other conditions with neurological impairment; four are prospective studies while one a retrospective review.

**Methods:**

this is a case report of eighteen children (sex ratio 11/8, median age 17 months, range 2–36 months) under three years of age, followed by a multidisciplinary team at the Bambino Gesù Children Hospital. The median follow-up was of 31.5 months (range 1–69 months). Response to treatment was assessed according to the Drooling Impact Scale administered at time 0 and after 1 month. All patients have an important neurological impairment: nine patients have a cerebral palsy (Gross Motor Function Classification System class V) and nine a genetic/malformative syndrome. Twelve patients have a tracheostomy and two need mechanical ventilation. Gastrostomy is present in 16 out of 18 patients. All patients received Glycopirrolate. The median starting daily dose was 0.065 mg/kg/die (range 0.02–0.21 mg/kg/die) three times a day. The drooling impact scale was administered at time O and after 1 month.

**Results:**

Four out 18 patients stopped treatment for adverse event, lack of efficacy or parental decision. The mean Drooling Impact Scale at time 0 was 89 (range 81–100) and after 1 month 61(range 43–78); the difference was statistically significant (*P* < 0.001). The overall response to treatment was 94%.

**Conclusions:**

This is the first study to determine the safety and effectiveness of Glycopyrrolate in decreasing drooling in a specific subset of patients. No major side effects were observed. Further comparative studies are needed to confirm our results.

## Introduction

Drooling is the involuntary loss of saliva and oral content; the salivary continence was normally reached by age of 15–18 months in developmentally normal children [1]. The drooling persistence is common in children with neurological disorders and impairment -as cerebral palsy- due to an oral motor dysfunction, dysphagia, and/or intraoral sensitivity disorder [1]. Management of drooling is a major concern in this population based on physical, behavioral, medical and surgical treatments individually tailored [1, 2]. The medical treatment is based on drugs that reduce the volume of saliva. Being the salivary glands controlled by the parasympathetic autonomic nervous system, the anticholinergic drugs are administered to reduce salivary flow [2].

Glycopyrrolate (GLY) is an anticholinergic agent with an oral bioavailability, competitively inhibiting acetylcholine receptors on peripheral tissues, reducing salivation rate [3–5]. GLY is currently the only oral formulation of an anticholinergic drug approved by the United States Food and Drug Administration (FDA) to treat drooling in children aged 3–16 years [1]. As an anticholinergic, GLY has several systemic effects namely gastrointestinal, genitourinary, cardiovascular, respiratory and ophthalmic side effects [3–8]. Any data are available on pediatric population under 3 years of age. The aim of this case report is to report the safety and efficacy of GLY in a subset of children with severe neurological impairment also called children with medical complexity (CMC) [ 9, 10] who started the treatment before 3 years of age.

## Patients and methods

All patients were followed by a multi-disciplinary team at the Bambino Gesù Children Hospital. Eighteen children (see Tab 1) with medical complexity who were younger than 3 years of age, received GLY treatment in off label setting. The response to treatment was assessed according to the Drooling Impact Scale at time O and time + 1 [11]. The medical records were reviewed for this study, in particular diagnosis and the functional impairment. All patients presented a severe neurological impairment and can be considered CMC. The patient characteristics were resumed in Table 1. Nine patients have a cerebral palsy (Gross Motor Function Classification System class V) while nine patients presented a genetic or malformative syndrome. Twelve patients have a tracheostomy and 2 need mechanical ventilation. Gastrostomy is present in 16 out of 18 patients. The median starting daily dose was 0.065 mg/kg/die (range 0.02–0.21 mg/kg/die) in three times a day, reached a median dose of 0.07 mg/kg/die (range 0.02–0.28 mg/kg/die); in 14 patients the starting dose was not modified.

**Table 1:** Patients charactheristics

Patients	18
Age	
Median	17.5 months
Range	2–36 months
< 12 months	6 patients
Sex	
Male	10
Female	8
Weight	
Median	9.8 kg
Range	3.5–22 kg
Diagnosis	
CEREBRAL PALSY	9
Genetic/Malformative	9
COGNITIVE IMPAIRMENT	100%
Speech Anomalies	100%
Gastrostomy	
Yes	16
No	2
Tracheostomy	
Yes	12
No	6
Mechanical Ventilation	
Yes	2
No	16

## Results

The mean Drooling Impact Scale at time 0 was 89 (range 81–100) and after 1 month 61 (range 43–78); the difference was statistically significant (*P* < 0.001). Considering patients who presented a decrease in the Drooling Impact Scale after 1 month, the overall response to treatment was 94%.; one patient stopped treatment soon after the first month for lack of efficacy while in two patients the treatment was discontinued after 6 months after medical and parental decision to perform the salivary duct ligation. In one patient, treatment was discontinued after 9 months for urinary retention that had no clear relationship with GLY administration.

In 10 patients, video fluoroscopy for swallowing or a gastric scintigraphy was performed before the GLY administration confirming a chronic lung aspiration. GLY was the first medical treatment for drooling in 17 out of 18 patients; one patient received intradermal scopolamine before GLY that was discontinued for toxicity. At median follow-up of 31.5 (range 1–69 months) from starting treatment, 14 patients continue the GLY adjusting the dose according to the weight gain.

all the participants and their parents gave informed consent before starting the experimental sessions. The procedure was approved by Ethics Committee of Bambino Gesù Children’s Hospital (Rome, Italy).

## Discussion

CMC may have a congenital or acquired disease with a severe neurologic and functional impairment and with a complete dependence for daily life activities; the presence of tracheostomy with or without mechanical ventilation, the presence of gastrostomy for enteral feeding, of a central venous access as the need of frequent aspirations of saliva during the day characterized this population [9, 10]. Especially in this subset of patients, the drooling control could represent a major goal in their clinical management. In fact, the drooling represents a major issue in patients with neurological impairment for its clinical and social impact. These children are at risk of saliva aspiration which can cause recurrent pneumonia and impairment in the gastric acid reflux removal related with esophageal dysmotility and esophagitis. The accumulation of saliva in the mouth can increase oral infections and damage the teeth. Moreover, the halitosis has a bad impact on social relationship.

Medical treatment is based on anticholinergic drugs as transdermal scopolamine, benzotropine and GLY [2–8, 12, 13]. GLY (Glycopyrrolate or glycopyrronium bromide) is a synthetic quaternary ammonium anticholinergic agent with poor blood–brain barrier penetration and consequently has limited and rare central effects. GLY was approved for clinical use in 1961 for peptic ulcer disease in adults. Actually, the oral GLY formulation was approved by the United States Food and Drug Administration (FDA) to treat drooling in children aged 3–16 years [3–14]. Orally GLY has relatively low and variable bioavailability influenced by high-fat meal that significantly decreased the absorption and excreted largely via the kidneys. After oral administration, GLY half-life is about 3 h and was present in plasma for < 12 h, following oral administration [14]. The suggested daily dosage is 0.02–0.1 mg/kg in 3 daily dose [ 3–8].

Five studies reported on GLY use for the treatment of drooling in children with cerebral palsy and other conditions with neurological impairment; four are prospective studies [5, 6, 8, 15, 16] while one a retrospective review [7]. Different dosages were proposed but almost the daily dosage was fractionated over three doses with limited toxicities and efficacy in reducing drooling in a mixed population including children with more than 3 years of age and young adults. In the randomized double-blind, dose-ranging trial [8], 39 children were treated according to two dosage regimens based on weight. Children less than 30 kg were started at 0.6 mg, with weekly increases to 1.2 mg, 1.8 mg, and 2.4 mg, whereas children over 30 kg were started at 1.2 mg, with weekly increases to 1.8 mg, 2.4 mg, and 3.0 mg. In the most recent prospective randomized placebo-controlled phase III study [15], GLY resulted to be significantly superior to placebo in reducing drooling in children aged 3–16 years affected by cerebral palsy or other neurological conditions associated with drooling problem. The dose used ranged from 0,02 mg/kg to 0,1 mg/kg accounting of the wide oral bioavailability range.

We present a case report of CMC treated with GLY before 3 years of age and with a median weight of 9.8 kg (range 3.5–22 kg). GLY seems effective in decreasing drooling with an overall response of about 94% with a statistically significant reduction of mean value of Drooling Impact Scale considered between baseline and follow-up (*P* < 0.001). The treatment was well tolerated with limited toxicities and a prolonged administration, mean treatment duration was of 31.5 months (range 1–69 months).

The median daily starting dose of 0.06 mg/kg (range 0.02–0.21 mg/kg) up to 0.07 mg/kg (range 0.02–0.28 mg/kg) in 3 daily dose with a high dose/kg for low weight patients due to the dosage adjustment using the 0.5 mg tablets. In fourteen patients, the starting dose controlled drooling without no need of dose/kg increase. We observed a side effects occurrence of about 5%; probably the limited side effect occurrence could be related to the young age of patients. It should be also considered that the most frequent side effects of GLY are dry mouth and thick secretions, both difficult to report in this age group.

Although our experience is limited, this is the first prospective case report reporting on the GLY use for drooling control in a population of CMC with severe neurological impairment under 3 years of age. An objective measure was used to confirm efficacy of the treatment. GLY is an effective drug and can be used without major complications in this age group for a long period with important social and medical implications. Further prospective and comparative studies are needed to confirm the safety of GLY and its efficacy in younger population. Moreover, the highly variable pharmacokinetics should be considered in a prospective study.

## Data Availability

No datasets were generated or analyzed during the current study.

## Abbreviations

FDA: Food and Drug Administration
GLY: Glycopyrrolate
CMC: children with medical complexity
